# Primary sclerosing cholangitis and the risk of colon neoplasia in patients with Crohn’s colitis

**DOI:** 10.1093/gastro/gov007

**Published:** 2015-02-26

**Authors:** Udayakumar Navaneethan, Tarun Rai, Preethi GK Venkatesh, Ravi P Kiran

**Affiliations:** ^1^Department of Gastroenterology, Digestive disease Institute, The Cleveland Clinic, Cleveland, OH, USA; ^2^Department of Colorectal Surgery, Digestive disease Institute, The Cleveland Clinic, Cleveland, OH, USA

**Keywords:** Crohn’s colitis, primary sclerosing cholangitis, colon neoplasia

## Abstract

**Background and aim:** Crohn’s colitis (CC) is associated with primary sclerosing cholangitis (PSC). However the risk of colon cancer or dysplasia in CC and PSC is unclear. Our aim was to study the risk of colon neoplasia in CC in patients with and without PSC.

**Methods:** This is a nested, case-control cohort study of all patients diagnosed with concurrent CC and PSC, seen at the Cleveland Clinic between 1985 and 2012. Forty-three patients with both CC and PSC were compared with a random sample of 159 CC controls without PSC during the same period.

**Results:** Seven (16.3%) of 43 CC patients with PSC developed colon cancer or dysplasia, compared with 22 (13.8%) of 159 controls (*P = *0.98). Of seven colon neoplasia cases in the PSC group, 100% occurred proximal to the splenic flexure, compared with 50% (11/22) cases of colon neoplasia in controls occurring in the proximal colon (*P = *0.001). Based on Cox regression analysis, male gender independently increased the risk of neoplasia [hazard ratio (HR) = 2.68; 95% confidence interval (CI) 1.30–5.54; *P = *0.008], as did age at CC diagnosis (HR = 1.29; 95% CI 1.14–1.47; *P* < 0.001), while the use of azathioprine/6-mercaptopurine was protective (HR = 0.30; 95% CI 0.13–0.70; *P = *0.005). The presence of PSC did not increase the risk for colon neoplasia (HR = 0.45; 95% CI 0.18–1.13; *P = *0.09).

**Conclusions:** CC patients with PSC appear not to be at increased risk of developing colon neoplasia. Among patients in our cohort with colon neoplasia and concurrent PSC, the neoplasia occurred in the proximal colon in all cases.

## Introduction

Primary sclerosing cholangitis (PSC) is a hepatobiliary disorder commonly seen in patients with underlying inflammatory bowel disease (IBD), most commonly ulcerative colitis (UC) [1–3]. In the presence of concurrent PSC, UC patients present with a higher prevalence of rectal sparing, backwash ileitis and colorectal neoplasia [4–8]. Patients with Crohn’s colitis (CC) who are also affected by PSC are a smaller group ranging between 1.3% and 14% [3].

Patients with IBD are prone to an increased risk of colorectal neoplasia, especially when they have had IBD for more than 10 years, early onset of the disease and extensive involvement of the colonic mucosa [9]. Several studies have shown that, in patients with UC, simultaneous PSC increases the risk of developing colitis-associated colorectal cancer (CRC) [10–14]. The 10-year and 20-year risks of developing colorectal neoplasia have been reported as 14% and 31%, respectively [12]. In patients with both CC and PSC, this risk is less clear. In a study from Europe, CC increased the risk of colon neoplasia [15]. However another study from England comparing CC patients with and without PSC, CC did not seem to increase the risk for neoplasia of the colon [16]. In our study, comparing UC and Crohn’s disease (CD) patients with simultaneous PSC, PSC+CD patients had a lower risk of colon neoplasia when compared with patients with PSC+UC [17].

To our knowledge, the risk of colon neoplasia in patients with CC and concurrent PSC—in comparison with patients with CC alone—has not been investigated in North America. The aim of our study was to evaluate and describe the clinical characteristics and to determine the risk of colorectal neoplasia in a cohort of patients with PSC+CC in comparison with a group of patients with CC without PSC.

## Patients and Methods

### Patients

The historical cohort study was approved by the Cleveland Clinic Institutional Review Board. We have previously described the manner in which the database was set up and populated; this was a retrospective database study [17–19]. We identified 43 patients with both CC and PSC up to 2012. We included all patients older than 18 years, with PSC concurrent with CD.

The control group was randomly selected from a larger population of patients with CC who did not have associated PSC during the same study period. Selection was performed using a random number table for the last two digits of the 8-digit medical record number. Medical record numbers are assigned consecutively at first clinic encounter. Patients with indeterminate colitis and patients who did not have follow-up at the Cleveland Clinic were excluded.

PSC was defined as the presence of intra- and/or extra-hepatic bile duct abnormalities in the form of beading, duct ectasia, and stricturing of the intra- or extra-hepatic bile ducts from endoscopic retrograde cholangiopancreatography, and/or magnetic resonance cholangiopancreatography [1]. The diagnosis of CC in PSC was made as previously described [17, 18].

### Demographic and clinical variables

The following demographic and clinical variables were obtained from patients’ medical records: age, gender, smoking and alcohol history, family history of IBD, PSC, or colon cancer in first degree relatives, duration and severity of PSC at diagnosis or earliest available calculated based on the Mayo PSC risk score [20], development of colon neoplasia and cholangiocarcinoma during the follow-up period, outcomes (patient alive at last follow-up, dead or had liver transplantation), the use of long-term medical therapy including corticosteroids, immunomodulators including azathioprine/6-mercaptopurine, biologics and ursodeoxycholic acid.

Disease activity in CD patients was assessed endoscopically using the CD Index of Severity, based on colonoscopy at diagnosis or the earliest available colonoscopy at our institution.^21^ In cases where this information was not available—particularly in patients who had undergone colonoscopy prior to 1998 and colonoscopy performed by non-IBD physicians, the endoscopist’s reported assessment was used. A disease flare of UC or CD was defined by the presence of clinical symptoms requiring a short course of corticosteroids.

Our study patients underwent colonoscopic surveillance for the development of colonic neoplasia or colon cancer every 1–2 years. Information on the presence of dysplasia was obtained from the pathology reports. Colonic neoplasia located proximally to the splenic flexure was classified as proximal and, if located distally to the splenic flexure, was classified as distal.

## Outcome Measurement

The primary outcome of interest was the development of colon cancer or dysplasia. The secondary outcome was to identify the risk factors that predict the development of colon neoplasia during follow-up in patients with CC.

## Statistical Analysis

Descriptive statistics were computed for all factors. These include medians, 25^th^ and 75^th^ percentiles, range, or mean and standard deviation for continuous factors, and frequencies and percentages for categorical factors. Wilcoxon’s rank sum tests for continuous factors and Pearson’s chi-squared or Fisher’s exact tests for categorical factors were used.

Patients were analysed from the onset of CC to an outcome or censor. Censoring was performed at the time of colectomy, last colonoscopy, or death. Kaplan-Meier survival curves were constructed to calculate the rates of development of dysplasia or cancer, and curves were compared between PSC+CC patients and CC controls using the Log-rank test. A Cox proportional hazards model was used to quantify the risk of PSC for dysplasia or cancer, to identify risk factors, and to adjust for possible confounding variables, such as extent and duration of disease, age, sex, and cigarette smoking. A significance level of 0.05 was used for all analyses. All analyses were performed using R 2.10.1 software (The R Foundation for Statistical Computing, Vienna, Austria).

## Results

### Demographic and clinical characteristics

Basic demographic and clinical information, including age, sex, race, and colonoscopic extent of CC, are summarized in Table 1. Basic demographics were similar in both the groups. Twenty-one (48.8%) patients in the PSC+CC group underwent orthotropic liver transplantation (OLT) on follow-up. The median age at the time of OLT was 45 years.

**Table 1.** Comparison of demographic and clinical variables between CC patients with PSC and CC controls without PSC

Variable	PSC with CC *n = *43	CC alone *n = *159	*P*-value
Age at diagnosis of CC (years, mean ± SD)	29.6 ± 14.5	28.7 ± 13.6	0.75
Male (*n*, %)	17 (39.5%)	86 (54.1%)	0.09
Body mass index [g/m^2^, median (interquartile range)]	26.7 (21.8–28.9)	25.2 (22.3–29.4)	0.85
Smoker (*n*, %)			0.03
Yes	5 (11.6%)	24 (15.1%)	
Ex-smoker	5 (11.6%)	50 (31.4%)	
Alcohol (*n*, %)			<0.001
Yes	6 (14.0%)	64 (40.3%)	
Ex-alcohol user	2 (4.7%)	0 (0%)	
Age at diagnosis of PSC (years, mean ± SD)	37.5 ± 13.3	N/A	N/A
Bile duct involvement		N/A	N/A
Intrahepatic only	10 (23.3%)		
Extrahepatic only	1 (2.3%)		
Intra- and extrahepatic	32 (74.4%)		
Initial PSC Mayo risk score [median (range)]	1.19 (-1.60–3.43)	N/A	N/A
Liver transplantation during follow-up (*n*, %)	21 (48.8%)	N/A	N/A
Number of CC flares (mean ± SD)	1.9 ± 0.6	1.3 ± 0.9	0.006
Endoscopic disease activity at diagnosis (moderate-to-severe)	4 (9.4%)	100 (62.9%)	<0.001
Ursodexoxycholic acid use (*n*, %)	38 (88.4%)	0 (0%)	<0.001
Azathioprine/6-mercaptopurine use (*n*, %)	6 (14%)	88 (55.3%)	<0.001
Biologics use (*n*, %)	4 (9.4%)	68 (42.8%)	<0.001
Colectomy during follow-up (*n*, %)	15 (34.9%)	112 (70.5%)	0.11
Age at colectomy [years,median (interquartile range)]	49 (32.5–52.5)	34 (26–51.5)	0.11
Colon dysplasia (*n*, %)			1.0
Low-grade	2 (4.7%)	9 (5.7%)	
High-grade	2 (4.7%)	9 (5.7%)	
Colon cancer (*n*, %)	3 (6.9%)	4 (2.5%)	0.17
Colon dysplasia and/or cancer (*n*, %)	7 (16.3%)	22 (14.8%)	0.98
Cholangiocarcinoma (*n*, %)	1 (2.4%)	0 (0%)	N/A

CC = Crohn’s colitis; SD = standard deviation; N/A = not available; PSC = primary sclerosing cholangitis

### Clinical phenotype of Crohn’s Colitis

All patients with CC concurrent with PSC had colon involvement, with nine patients having additional small bowel involvement. Thirty-four patients with isolated colon involvement alone had extensive colitis; granulomas were seen in 23 patients, fistulas in 2 patients, while patchy colitis with deep ulcers was seen in the remaining 9 patients. Among these nine, there was continuous inflammation affecting the entire right colon, starting from the splenic flexure and extending to the cecum, with patchy colitis seen in the left side. The mean number of flares was higher in the PSC+CC group than in CC alone (1.9 ± 0.7 *vs.* 1.3 ± 0.9; *P = *0.006).

### Colon neoplasia

Seven (16.3%) of 43 CC patients with PSC developed CRC or dysplasia, compared with 22 (13.8%) of 159 controls (*P = *0.98). Of seven cases of colon neoplasia in the PSC group, 100% occurred proximally to the splenic flexure, compared with 11/22 (50%) cases of proximal colon neoplasia in the controls (*P = *0.001). In the PSC+CC group, high-grade dysplasia was seen in two patients, low-grade dysplasia in two and colon cancer in three patients while, in the CC-alone group, nine had low grade dysplasia, nine had high grade dysplasia and four had colorectal cancer (Table 1). Among the seven patients with colon neoplasia with PSC and CC, three had flat dysplasia, while one patient with high grade dysplasia had a lesion that could not be removed endoscopically. All the three patients with colon cancer had polypoid mass/lesion.

### Surgery for Crohn’s Colitis

One hundred and twelve patients in the CC-alone group underwent colectomy, in contrast to 15 patients in the PSC+CC group. Among the 15 patients in the PSC+CC group, 8 underwent colectomy for steroid refractory/dependent disease, while the other 7 patients underwent colectomy for colon cancer/dysplasia. Among the 112 patients who underwent colectomy in the CC-alone group, 90 underwent colectomy for steroid refractory/dependent disease, while the other 22 patients underwent colectomy for colon cancer/dysplasia.

### Risk factors of colon neoplasia

**Figure 1.**
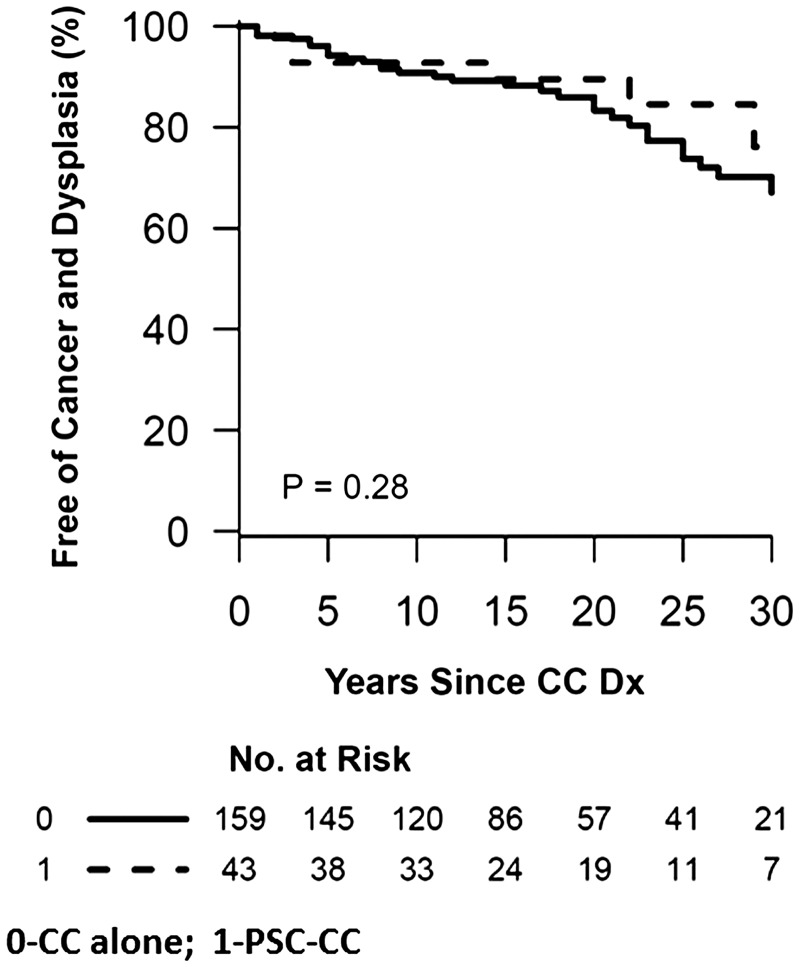
Kaplan-Meier curve of the proportion of patients free from colon neoplasia among CCpatients with PSC and CC controls without PSC. There was no significant different between the groups (Log-rank *P = *0.28).

Figure 1 summarizes the Kaplan-Meier curve of the proportion of patients free of colorectal cancer or dysplasia among CC patients with PSC and CC controls without PSC. There was no significant different between the groups (*P = *0.28).

**Figure 2.**
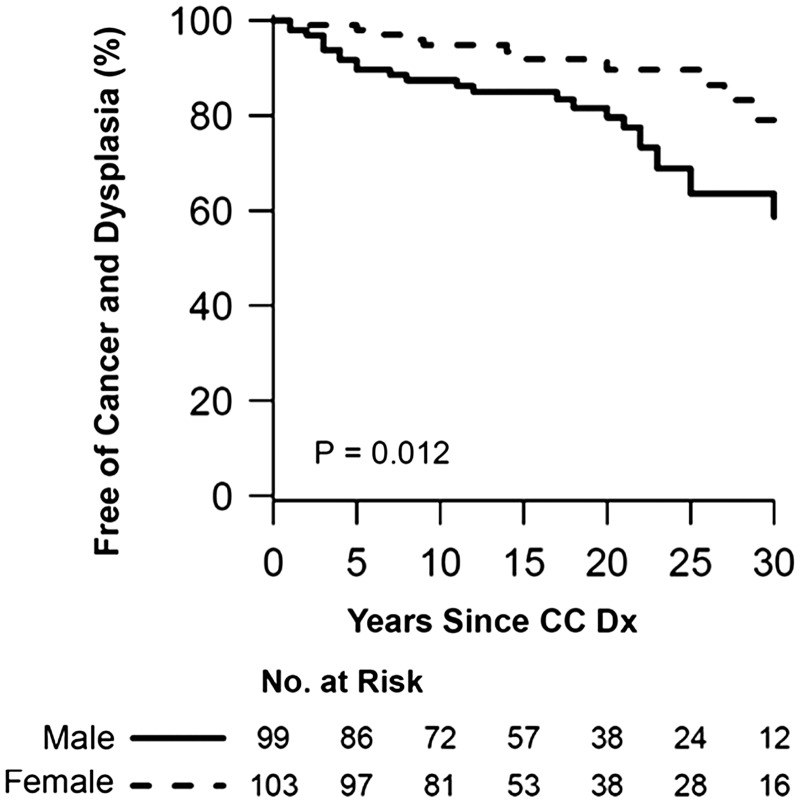
Kaplan-Meier curve of the proportion of CC patients free from colon neoplasia based on gender. Male patients were less likely than females to be free from colon neoplasia (Log-rank *P = *0.012).

**Figure 3:**
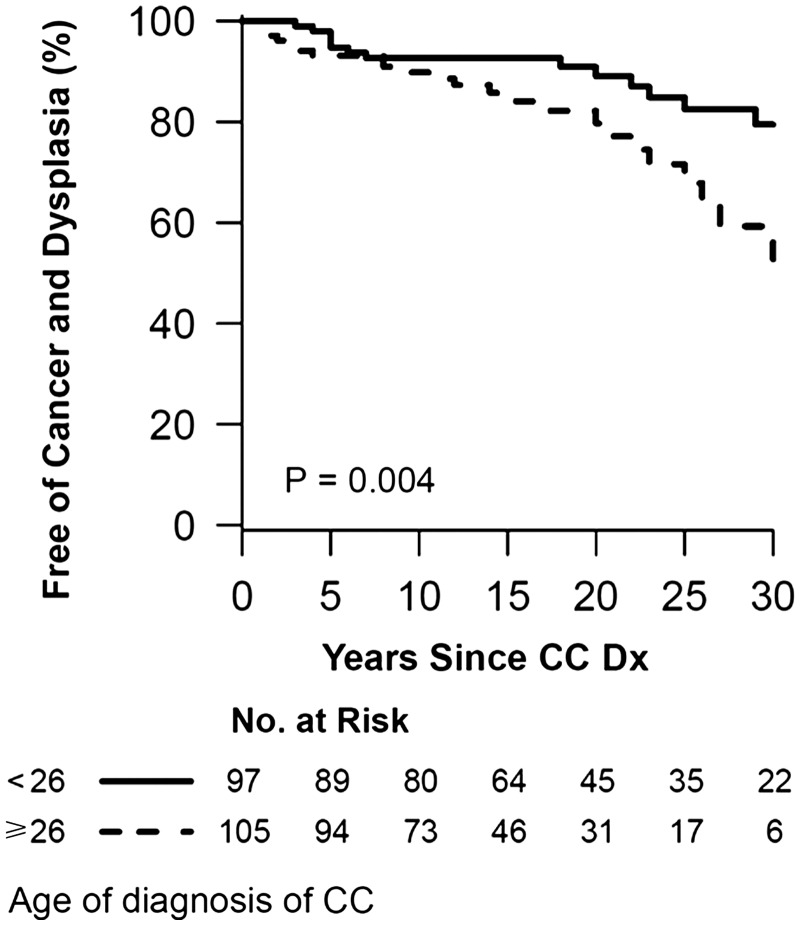
Kaplan-Meier curve of the proportion of patients free of colon neoplasia based on age at diagnosis of CC. Patients with age greater than 26 years at CC diagnosis were less likely to be free from colon neoplasia than patients under 26 years (Log-rank *P = *0.004).

Based on Cox regression analysis, male gender independently increased the risk for neoplasia [hazard ratio (HR) = 2.68; 95% confidence interval (CI) 1.30–5.54; *P = *0.008], as does age at CC diagnosis (HR = 1.29; 95% CI 1.14–1.47; *P* < 0.001), while the use of azathioprine/6-mercaptopurine was protective (HR = 0.30; 95% CI 0.13–0.70; *P = *0.005). The presence of PSC did not increase the risk for neoplasia (HR = 0.45, 95% CI 0.18–1.13; *P = *0.09) (Table 2). Figures 2 and 3 summarize the Kaplan-Meier curves of the proportions of patients free from colon neoplasia, based on gender and age at diagnosis of CC.

**Table 2.** Cox proportional hazards analysis for colon dysplasia/cancer

Variable	Hazard ratio (95% CI)	*P*-value
Male	2.68 (1.30–5.54)	0.008
Age at Crohn’s colitis diagnosis (per 5-year increase)	1.29 (1.14–1.47)	<0.001
Extensive colitis	1.10 (0.53–2.26)	0.80
Presence of primary sclerosing cholangitis	0.45 (0.18–1.13)	0.09
Use of azathioprine/6-mercaptopurine	0.30 (0.13–0.70)	0.005
Family history of colon cancer	1.52 (0.64–3.67)	0.34
Smoker (current or past)	0.59 (0.29–1.22)	0.16
Moderate or severe disease activity at diagnosis	0.89 (0.45–1.77)	0.75

CI = confidence interval

### Discussion

To our knowledge, the clinical outcome of CC in patients with concurrent PSC has not been compared against patients with CC alone. Our study—the first of its kind from North America—showed that PSC did not increase the risk of colon neoplasia in patients with CC. All instances of colon neoplasia in the PSC group occurred in the proximal colon. We also identified that male gender and age at CC diagnosis independently increased the risk for neoplasia.

Patients with UC are at an increased risk of colorectal neoplasia if they have had the disease for more than 10 years, if they were younger at the time of onset of the disease and if there is extensive involvement of colonic mucosa [9]. Some studies have shown an increased risk of colon carcinoma in patients with CD [22–24], while others refute the possibility that CD is associated with an increased risk of colon carcinoma [25–28]. However, in these studies, if duration of disease is taken into account, the risk of colon cancer is increased.

The risk of colon cancer/dysplasia in the presence of PSC has also been investigated in both UC and CD. Several studies have shown that, in patients with UC, concurrent PSC increases the risk of developing colitis-associated CRC [10–14]. However it is unclear whether PSC also increases the risk of developing colon neoplasia in patients with CC. In a study that compared CD patients with and without PSC, colonic CD did not seem to increase the risk for neoplasia of the colon [16]. We previously reported that patients with PSC+CD appeared to have a lower risk of neoplasia than patients with PSC+UC [17]. In the present study, we observed that, the cumulative occurrence of colon cancer/dysplasia was not influenced by the presence of concurrent PSC in CC patients.

The low risk of colon neoplasia in patients with PSC and CC contrasts with a previous report in which 2/9 patients with PSC and colonic Crohn’s disease developed colon cancer over a period of 15 years [29]. In fact, a recent population-based cohort study from Denmark showed that the risk of colon neoplasia in CC is not increased and that the risk of colon neoplasia appears to be decreasing [30]. The authors propose that the use of newer treatments is probably associated with decreased risk. Also, we found that the use of azathioprine was associated with a lower risk of colon neoplasia, supporting these observations.

We earlier showed that right-sided cancers predominate in PSC patients who have concurrent UC and that the duration and severity of PSC does not appear to be related to the risk of colon neoplasia [31]. Altered bile acid metabolism is postulated to be responsible for the predominance of right-sided colon neoplasia in CC patients with PSC.

Our study is clinically significant for a number of reasons: we found that the risk of colon neoplasia in patients with both CC and PSC appears to be similar to the risk in patients with CC alone. Also the disease behavior in patients with PSC appears to be similar and CC presents with colonic disease with occasional small bowel involvement and mimics UC in most cases.

There are certain limitations of our study. The study population was recruited from a sub-specialty tertiary care referral center. This contributed to a referral bias. We observed that 14.8% of CC controls had colon neoplasia, which is higher than previously reported. Some patients were referred to our institution with a diagnosis of neoplasia, which could also contribute to the high rate of colon neoplasia. Nevertheless, this is one of the largest studies of the natural histories of CC and PSC, in which PSC did not increase the risk of colon neoplasia in patients with CC.

To conclude, CC patients with simultaneous PSC appear not be at increased risk of developing colon neoplasia when compared with CC alone. In all patients in our cohort with colon neoplasia and concurrent PSC, the neoplasia occurred in the proximal colon.

## Funding

The study is supported by a research grant from the inflammatory bowel disease working group (U.N.)

*Conflict of interest statement:* none declared.
